# Prediction of drug–drug interactions between roflumilast and CYP3A4/1A2 perpetrators using a physiologically-based pharmacokinetic (PBPK) approach

**DOI:** 10.1186/s40360-023-00726-2

**Published:** 2024-01-02

**Authors:** Guangwei Jia, Congcong Ren, Hongyan Wang, Caixia Fan

**Affiliations:** 1 Department of pharmacy Liaocheng People’s Hospital, 252000 Liaocheng, Shandong Province China; 2 Center for Clinical Pharmacology Linyi People’s Hospital, Wuhan Road and Wo Hu Shan Road, 276000 Linyi, Shandong Province China

**Keywords:** Roflumilast, PBPK model, Dosing regimen adjustment, DDI simulation

## Abstract

This study aimed to develop a physiologically-based pharmacokinetic (PBPK) model to predict changes in the pharmacokinetics (PK) and pharmacodynamics (PD, PDE4 inhibition) of roflumilast (ROF) and ROF N-oxide when co-administered with eight CYP3A4/1A2 perpetrators. The population PBPK model of ROF and ROF N-oxide has been successfully developed and validated based on the four clinical PK studies and five clinical drug-drug interactions (DDIs) studies. In PK simulations, every ratio of prediction to observation for PK parameters fell within the range 0.7 to 1.5. In DDI simulations, except for tow peak concentration ratios (C_max_) of ROF with rifampicin (prediction: 0.63 vs. observation: 0.19) and with cimetidine (prediction: 1.07 vs. observation: 1.85), the remaining predicted ratios closely matched the observed ratios. Additionally, the PBPK model suggested that co-administration with the three perpetrators (cimetidine, enoxacin, and fluconazole) may use with caution, with CYP3A4 strong inhibitor (ketoconazole and itraconazole) or with dual CYP3A41A2 inhibitor (fluvoxamine) may reduce to half-dosage or use with caution, while co-administration with CYP3A4 strong or moderate inducer (rifampicin, efavirenz) should avoid. Overall, the present PBPK model can provide recommendations for adjusting dosing regimens in the presence of DDIs.

**Supplementary Information:**

The online version contains supplementary material available at 10.1186/s40360-023-00726-2.

## Introduction

Chronic obstructive pulmonary disease (COPD) is a progressive respiratory condition characterized by persistent airflow limitation and respiratory symptoms [1]. It is a leading cause of chronic morbidity and mortality worldwide, accounting for approximately global prevalence of 11.7% [2] and 5% of all deaths worldwide [3]. Cyclic adenosine monophosphate (cAMP) has been associated with COPD for several decades [4]. Phosphodiesterase-4 (PDE-4) is an enzyme that plays a crucial role in regulating cAMP levels within cells [4, 5]. PDE4 has been studied extensively and identified as a promising therapeutic target for COPD for many years [6].

Roflumilast (ROF) is a first selective PDE-4 inhibitor indicated as a treatment to reduce the risk of COPD exacerbations [7]. Clinical use has been approved by FDA for once-daily (OD) administration of 0.25 mg and 0.5 mg tablets [8]. *In vitro*, ROF is primarily metabolized by cytochrome P450 (CYP) enzymes, specifically CYP1A2 and CYP3A4, leading to the formation of approximately 10 metabolites [9]. Among these metabolites, roflumilast N-oxide (ROF N-oxide) is the most important active metabolite, exhibiting comparable activity to ROF and approximately 11 times higher plasma exposure [9]. While ROF itself inhibits 10% of PDE4, *in vivo*, 90% of PDE inhibition is contributed by ROF N-oxide [9].

In clinic practice, patients may be treated with the concurrent use of multiple drugs, which put them at the risk of drug-drug interactions (DDIs) [10]. Furthermore, pharmacokinetic (PK)-related and pharmacodynamics (PD)-related DDIs are vital considerations in clinical practice as they can affect the efficacy and safety of drug therapy [11]. PK-related DDIs mediated by CYP enzymes have garnered significant attention over the past decades. In contrast, PD-related DDIs have received relatively little attention, despite the fact that their frequencies are approximately 1.9-fold higher than those of PK-related DDIs [12].

Physiologically-based pharmacokinetic (PBPK) modeling is a valuable tool used for simulating and predicting the PK of drugs in the human. Additionally, PBPK models were commonly utilized to evaluate the possibility of clinical PK-based DDIs involving multiple drugs [13], as well as PD-related DDIs that had relatively few reported cases [12, 14]. By utilizing PBPK models, the magnitude and importance of DDIs can be predicted, thereby facilitating dose adjustments or the selection of alternative therapies to minimize the risk of adverse events [12, 14, 15].

Our objective was to develop a PBPK model to evaluate the impact of eight single and dual CYP3A4/1A2 perpetrators on the PK and PD (i.e., PDE4 inhibition) of both ROF and ROF N-oxide when co-administrated. Specially, the PBPK model was used to (i) predict the area under the curve (AUC) and maximum concentration (C_max_) of ROF and ROF N-oxide in healthy individuals and in COPD patients; (ii) predict PK- and PD-related DDIs of ROF when used in combination with eight CYP3A4 and CYP1A2 perpetrators, respectively; and (iii) recommend an optimal dosing regimen for DDIs. By employing the PBPK model, we aimed to enhance our understanding of how the co-administration of ROF and these perpetrators may influence the PK and PD. This information can ultimately guide dosage adjustments in the presence of DDIs.

## Methods

### PBPK model structures

As described in previous paper [12], the PBPK model consists of multiple compartments interconnected by blood flow rate, including the mucosa (gastro-intestine), blood (arterial supply and venous return), eliminating and non-eliminating tissues. The mucosa includes the duodenum, jejunum, ileum, cecum, colon, and rectum. The mucosa is characterized by its volume, gastric emptying time (with a mean of 15 min), small intestinal transit time (with a mean of 2.10 h), large intestinal transit time (with a mean of 44.20), as well as the mean pH of stomach (2.0) and different intestinal segments pH(ranging from 5.60 to 7.46). Each tissue compartment is defined by its volume, fraction of vascular and intracellular components, as well as pH (blood cells, interstitial fluid, intracellular fluid, and plasma). The distribution of ROF and ROF N-oxide is defined using the interstitial-to-plasma partition coefficient (K_Ins_,p) and intracellular-to-plasma partition coefficient (K_Inc_,p).

The human tissue distribution and cellular permeability of ROF and ROF N-oxide were described by Rodgers and Rowland, and the PK-Sim standard methods, respectively. The intrinsic unbound clearance (CL_int,u_) and plasma clearance (CL_P_) were estimated by Eqs. (1, 2 and 3) [16, 17].

1 $${\text{C}\text{L}}_{\text{i}\text{n}\text{t},\text{u}}=\frac{{\text{E}\text{n}\text{z}}_{\text{i}}}{{\text{I}\text{S}\text{E}\text{F}}_{\text{i}}\times {\text{e}\text{x}\text{p}\text{r}\text{e}\text{s}\text{s}\text{i}\text{o}\text{n}}_{\text{i}}}$$

Where CL_int,u_ (µL/min/pmol) is the unbound intrinsic clearance by the CYP3A4 or CYP1A2 enzyme; ${ \text{E}\text{n}\text{z}}_{\text{i}}$ represents metabolic the rate by the CYP3A4 or CYP1A2 enzyme (µL/min/mg); ISEF represents the intersystem extrapolation factor, with ISEF values of 0.33 and 0.39 for CYP3A4 and CYP1A2, respectively [18]. expression_i_ represents the abundance of CYP3A4 or CYP1A2 (137 and 52 pmol/mg protein for CYP3A4 and CYP1A2, respectively [18]). Enz_i_ was estimated using the following Eq. (2).

2 $${\text{E}\text{n}\text{z}}_{\text{i}}=\frac{{\text{V}}_{\text{m}\text{a}\text{x}}}{{\text{K}}_{\text{m}}+{\text{C}}_{\text{i}}}$$

Where V_max_ is maximal rate of metabolism by CYP3A4 or CYP1A2. K_m_ is the Michaelis-Menten constant for the conversion of ROF to ROF N-oxide. C_i_ is unbound ROF concentration. Values for V_max_ and K_m_ were available from the literature [9]. The relationship between CL_int,u_ and CL_P_ can be expressed as follows:

3 $${\text{C}\text{L}}_{\text{P}}={\text{R}}_{\text{b}\text{p}}\times \text{Q}\left(\frac{{\text{C}\text{L}}_{\text{i}\text{n}\text{t},\text{u}}}{{\text{C}\text{L}}_{\text{i}\text{n}\text{t},\text{u}}+\text{Q}\frac{{\text{R}}_{\text{b}\text{p}}}{{\text{f}}_{\text{u}\text{p}}}}\right)$$

Where R_bp_ is blood/plasma concentration ratio; Q is tissue blood flow, the average Q is 90 L/h in human [19]; f_up_ is fraction of unbound drug in plasma.

### Population PBPK model development

The PBPK model of ROF and ROF N-oxide was developed using PK-Sim (Version 10.0, Bayer Technology Services, Leverkusen, Germany) with the modelling parameters listed in Table 1. The modeling parameters were primarily taken form published papers [9, 20–22]. Some parameters were optimized to improve the description of human PK of ROF and ROF N-oxide. Log p was optimized to 3.5, providing a better fit to the observed C_max_ compared to the original value of 3.99 [9]. The K_Ins_,p scale for ROF was optimized to 5.0 to better describe the tissue distribution. However, a few parameters were not available in the literature, and were estimated by PK-Sim (R_bp_) or optimized (Log p of ROF N-oxide) by comparing the predicted and observed PK profiles.

**Table 1:** The used PBPK modelling parameters for ROF and ROF N-oxide

Parameters (Units)	Values		Source and comments
	ROF	ROF N-oxide	
MW(g·mol^−1^)	403.21	419.21	Chemspider
pKa (Base)	13.3 (acid); 2.4 (base)	12.92, 0.65	[9], Optimized for ROF N-oxide
LogP	3.5	2.6	Optimized
Solubility (μg·mL^−1^)	0.5 (@pH7.4)	-	[9]
P_app_ (X 10^–5^ cm⋅s^−1^)	0.01	-	[20]
f_up_	0.011	0.034	[9]
R_bp_	0.73	0.62	Calculated by PK-Sim
CYP3A4 CL_int,u_ (μL/min/mg)^a^	0.90/0.55	0.010/0.0093	Optimized based on the observed human PK
CYP1A2 CL_int,u_ (μL/min/mg)^a^	0.55/0.33	-	Calculated based on lower clearance in COPD patients
CL_a_ (μL/min/mg)^a^	-	0.10/0.093	
CL_R_(L/h)	GFR*f_up_		Default
GFR fraction	1.0		Default
K_Ins,p_ scale	5.0	-	Optimized based on the observed human PK
Partition coefficients	Rodgers and Rowland		Optimized based on the observed human PK
Cellular permeabilities	PK-Sim Standard		
Concentration (μM/L liver tissue)			
CYP3A4	4.32		Default
CYP1A2	1.80		
Abundance in HLM (pmol/mg protein)			
CYP3A4	137		[21]
CYP1A2	52		
k_deg_			
CYP3A4	0.019 h^−1^(liver),0.03 h^−1^ (intestine)		[22]
CYP1A2	0.017 h^−1^(liver), 0.03 h^−1^ (intestine)		
K_i_ CYP3A4 (μM)	2.79	-	[9]

-No data, *MW* Molecular weight, *pKa* Dissociation constant, *Log P* Lipophilicity, *P*_*app*_ Caco-2 cell permeability, *f*_*up*_ Unbound fraction in plasma, *R*_*bp*_ Blood-to-plasma concentration ratio, *CYP3A4/1A2 CL*_*int,u*_ unbound intrinsic clearance, *CL*_*a*_ Additional clearance, *CL*_*R*_ Renal clearance, *GFR fraction* Fraction of filtered drug in the urine, *GFR* Glomerular filtration rate, *K*_*Ins,p*_ interstitial-to-plasma partition coefficient, *k*_*deg*_ turnover of the metabolizing enzyme, *K*_*i*_ Concentration resulting in a 50% inhibition

^a^Values in healthy subjects and COPD patients, respectively

The metabolism of ROF to ROF N-oxide is primarily mediated by CYP1A2 and CYP3A4. In the PBPK model, the metabolism parameters are described by their CL_int,u_. Based on the HLM metabolism data in the paper [9], the CYP3A4 and CYP1A2 CL_int,u_ values for ROF were estimated as 45.7 (78.2% metabolism contribution [9]) and 11.8 µL/min/mg (20.2% metabolism contribution [9]), respectively, assuming a substrate concentration of 1.0 µM. Using Eq. (1), the CL_int,u_ (µL/min/pmol) values were calculated to be 1.01 and 0.58 µL/min/pmol, respectively. For the PBPK model, the final CL_int,u_ values for CYP3A4 and CYP1A2 were slightly optimized as 0.90 and 0.55 µL/min/pmol.

**Fig. 1:**
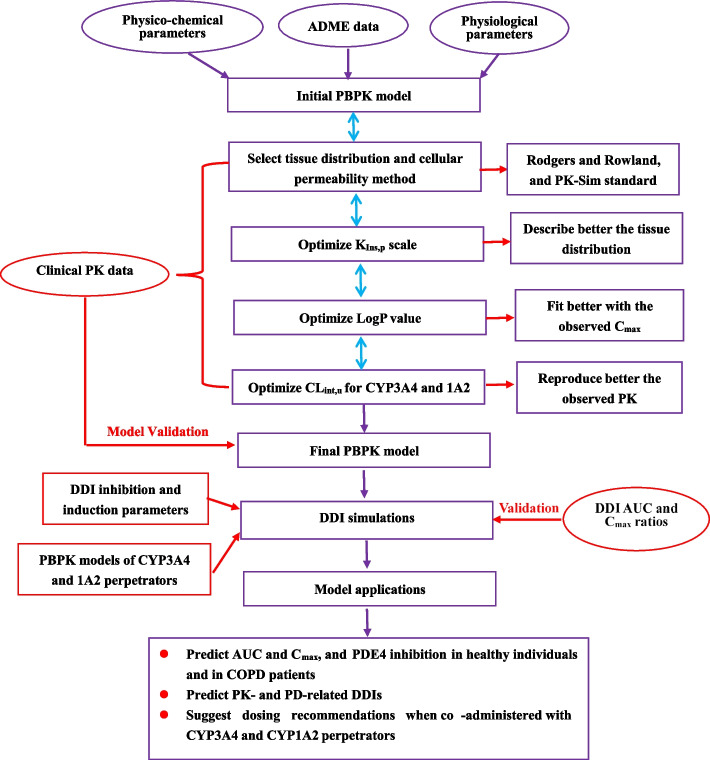
Workflow of ROF PBPK model development and validation

The Oral CL_p_ of ROF N-oxide was estimated to be an average value of 0.95 L/h [9]. ROF N-oxide is metabolized by CYP3A4, CYP1A1, and CYP2C9. In the PBPK model, the final CL_int,u_ value for CYP3A4-mediated metabolism of ROF N-oxide was optimized to 0.010 µL/min/pmol, and additional clearance (CL_a_, encompassing CYP1A1, and CYP2C9) was optimized to 0.10 mL/h/kg. Additionally, it has been reported that in COPD patients, PK exposures of ROF and ROF N-oxide are 60% and 8% higher, respectively, compared to healthy subjects. Consequently, in COPD patient population, the CL_int,u_ values for ROF mediated by CYP3A4 and CYP1A2 were decreased to 0.55 and 0.33 µL/min/pmol, respectively. Furthermore, the CL_int,u_ value for ROF N-oxide by CYP3A4-mediated metabolism and the CL_a_ in COPD patients were decreased to 0.0093 µL/min/pmol and 0.093 mL/h/kg, respectively. Moreover, there were no reports indicating the involvement of kidney transporters or tubules in the influx or efflux of ROF and ROF N-oxide. Therefore, the both fractions of GFR were set at 1.0. Figure 1 illustrates the workflow of ROF PBPK model development and validation.

### Population PBPK model validation

The four clinically observed PK profiles and data of ROF and ROF N-oxide in both healthy subjects and COPD patients were taken from the published papers [23–26] using Digit software (Version 1.0.4, Simulations Plus, USA). The developed PBPK model was validated by comparing the predicted PK profiles and data with the observed values. The prediction-to-observation ratios of AUC and C_max_ were calculated, and the commonly accepted criterion for these ratios is between 0.5 and 2.0. This criterion helps assess the accuracy of the model predictions by evaluating the agreement between the predicted and observed PK parameters.

### Population DDI simulations

The PBPK modeling parameters for eight CYP3A4 and CYP1A2 inhibitors and inducers are provided in Supplementary Table S1. The inhibition and induction parameters of these perpetrators against CYP3A4 and CYP1A2 enzymes are listed in Table 2 [27–32]. The PBPK model of ROF and ROF N-oxide was combined with the PBPK models of these eight perpetrators to simulate the effects of the perpetrators on the PK and PD (PDE4 inhibition) of ROF and ROF N-oxid. The total PDE4 inhibition values (tPDE4i) were calculated according to the following Eq. (4)

4 $$\text{t}\text{P}\text{D}\text{E}4\text{i}=\frac{{AUC}_{ROF}\times f_{up,ROF}}{{IC}_{50,ROF}\times\tau}+\frac{{AUC}_{ROF\;N-oxide}\times{fup,}_{ROF\;N-oxide}}{{IC}_{50,ROF\;N-oxide}\times\tau}$$

**Table 2:** The inhibition and induction parameters of CYP3A4 and CYP1A2 perpetrators

Perpetrators	CYP3A4 K_i_ (μM)	CYP1A2 K_i_ (μM)	EC_max_	EC_50_ (μM)
Ketoconazole (inhibitor, KET) [27]	0.0054	32		
Itraconazole (inhibitor, ITR) [28]	0.0013		-	-
Hydroxy-itraconazole [28]^a^	0.0023		-	-
Fluconazole (inhibitor, FLU) [29]	16.6		-	-
Fluvoxamine (inhibitor, FLUV)^b^	0.52	0.011	-	-
Enoxacin (inhibitor, ENO) [30]	-	110		
Cimetidine (inhibitor, CIM) [31, 32]	106	140.7		
Rifampicin (inducer, RIF) [28]	-	-	9.0	0.34
Efavirenz (inducer, EFA)^b^	-	-	5.2	0.07

^a^metabolite of itraconazole

^b^built in the PK-Sim

Where AUC_ROF_ and AUC_ROF N−oxide_ are AUC of ROF and ROF N-oxide (µg·h/mL), respectively; f_up,ROF_ and f_up,ROF N−oxide_ are free fraction of ROF and ROF N-oxide in plasma, respectively; IC_50,ROF_ and IC_50, N−oxide_ (µg/L) are concentration of ROF and ROF N-oxide resulting in 50% PDE4 inhibition *in vitro*, respectively; τ is dosing interval at repeated-doses (24 h). IC_50,ROF_ and IC_50, N−oxide_ are 0.3 and 0.8 µg/L in the literature [9].

The DDI simulations were verified by comparing the AUC and C_max_ ratios with and without perpetrators between predicted and observed, as reported in the referenced papers [27, 33–36]. In the DDI simulations, the dosage regimens for ROF and five perpetrators (KET, FLUV, RIF, ENO, and CIM) were determined based on the information provided in the published papers [27, 33–36]. However, for remaining three perpetrators (ITR, FLU, and EFA) for which no specific dosing information was available in the literature, the dosage regimens were set as follows: a single-dose of ROF at 0.5 mg OD with repeated-doses of ITR at 200 mg OD, FLU at 150 mg OD, and EFA at 600 mg OD for consecutive 14 days. Moreover, all DDI simulations were specifically conducted in healthy subjects.

### The demographic characteristics data

The demographic characteristics data used in the PBPK model were collected from the respective clinical studies. The virtual population information included age range, body weight, height, and the proportion of female participants. In cases where specific data was missing, the mean values provided in PK-Sim were used as a substitute.

## Results

### Validation of the population PBPK model for ROF and ROF N-oxide

**Fig. 2:**
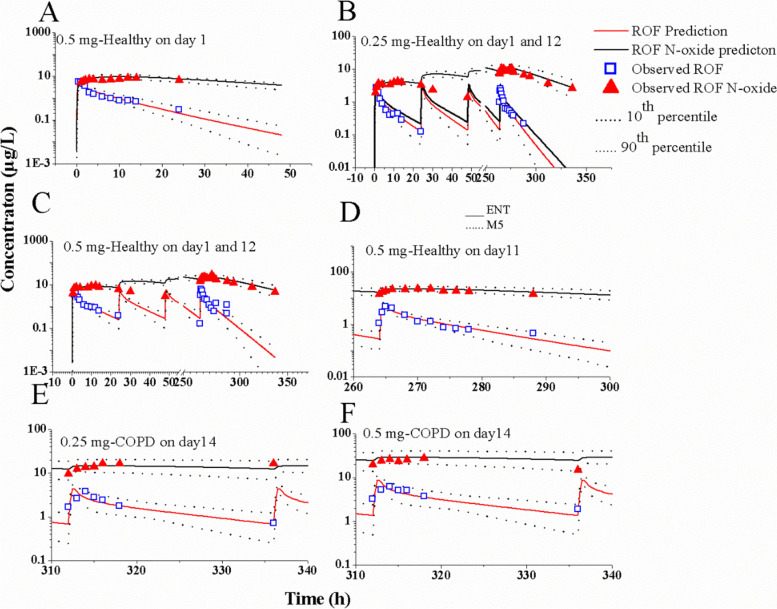
Simulations of plasma concentration-time profiles of ROF and ROF N-oxide after administration of single dose and repeated doses. The predicted and observed plasma concentration-time curves of ROF and ROF N-oxide in healthy subjects at a single dose of 0.5 mg (**A**), repeated doses of 0.25 mg (**B**) and 0.5 mg (**C**), repeated doses of 0.5 mg (**D**), as well as in COPD patients at repeated doses of 0.25 mg (**E**) and 0.5 mg (**F**). The blue open squares (RFO) and red solid up-triangles (RFO N-oxide) denote the clinically observed data

Figure 2 depicts the predicted and observed plasma concentration-time profiles in healthy subjects (Fig. 2A-D) and COPD patients (Fig. 2E/F) following oral administration of single or multiple doses at steady-state. The simulations suggest that the population PBPK model may agree well with the observed PK profiles of ROF and ROF N-oxide [23–26]. Table 3 presents the ratios of predicted and observed geometric mean AUC and C_max_, all of which range between 0.7 and 1.5. Notably, the majority of ratios fall within the range of 0.8–1.25. The simulations indicate the successful development of the population PBPK model, demonstrating its ability to accurately predict the AUC and C_max_ of ROF and ROF N-oxide at single and repeated doses in both healthy subjects and COPD patients.

**Table 3:** Predicted and observed geometric means of pharmacokinetic parameters of ROF and ROF N-oxide

Clinical studies	Drug	Dosage schedules (mg)	Subjects	AUC_0-last_ (μg·h/L, range)		C_max_ (μg/L, range)		Prediction/observation ratio	
				Prediction	Observation	Prediction	Observation	AUC	C_max_
Hauns et al. (2006) [23]	ROF	0.5, SD	Healthy	30.6 (16.2–44.8)	28.9 (22.9–36.3)	6.6 (4.6–12.8)	6.5 (5.0–8.5)	1.06	1.02
	ROF N-oxide			335 (241.5–352.3)	271.1 (217.3–338.1)	9.6 (7.5–16.5)	8.8 (7.1–11.0)	1.24	1.09
Bethke et al. (2007) [24]	ROF	0.25, RD, on day 1		14.2 (9.0–20.5)	18.1 (11.1–29.7)	2.8 (2.5–3.4)	2.9 (2.0–4.3)	0.78	0.97
	ROF N-oxide			182.9 (144.3–263.9)	178.6 (115.8–275.2)	4.1 (3.2–5.3)	4.5 (3.5–5.8)	1.02	0.91
	ROF	0.5, RD, on day 1		28.3 (18.1–41.1)	35.0 (20.5–59.8)	5.5 (4.5–6.9)	5.3 (4.2–6.6)	0.81	1.04
	ROF N-oxide			365.7 (288.6–527.4)	351.3 (235.5–524.0)	8.6 (6.6–11.0)	9.4 (7.5–11.8)	1.04	0.91
	ROF	0.25, RD, on day 12		15.6 (9.5–23.9)	17.0 (10.5–27.4)	3.0 (2.7–3.5)	3.1 (2.0–4.6)	0.92	0.97
	ROF N-oxide			250.2 (183.6–335.3)	179 (117.4–275.2)	11.6 (9.4–14.9)	10.5 (6.7–16.2)	1.40	1.10
	ROF	0.5, RD, on day 12		34.4 (19.9–55.6)	33.7 (19.3–58.7)	6.1 (5.0–7.6)	6.0 (3.8–9.6)	1.02	1.02
	ROF N-oxide			500.4 (367.1–670.7)	375.4 (231.5–608.7)	23.1 (17.9–29.7)	21.7 (13.9–33.9)	1.33	1.06
Mey et al. (2011) [25]	ROF	0.5, RD, on day 11		31.2 (19.0–47.8)	35.8 (27.8–46.1)	6.1 (5.0–7.6)	6.9 (4.9–9.6)	0.87	0.97
	ROF N-oxide			525.4 (385.5–704.2)	417 (299–582)	27.8 (21.5–35.7)	23.3 (17.3–31.3)	1.26	1.19
Facius et al. (2011) [26]	ROF	0.25, RD, on day 14	COPD	35.2 (16.5–65.3)	43.2 (NR)	4.5 (3.4–6.8)	3.8 (NR)	0.81	1.18
	ROF N-oxide			330.1 (4196.7–610.6)	416 (NR)	14.6 (8.9–26.3)	16.4 (NR)	0.79	0.89
	ROF	0.5, RD, on day 14		70.5 (62.6–130.7)	65.3 (NR)	6.7 (5.5–8.3)	6.2 (NR)	1.08	1.08
	ROF N-oxide			660.2 (393.3–1221.2)	510 (NR)	23.1 (17.9–29.7)	27.6 (NR)	1.29	0.84

*SD* Single-dose, *RD* Repeated-doses, *NR* not reported

### Sensitivity analysis

A sensitivity analysis was conducted on the optimized parameters of the PBPK model by varying each parameter by ± 100%. The results are presented in Supplementary Table S2. It was found that the AUC and C_max_ values for ROF were most sensitive to changes in Log P, with a sensitivity coefficient (SC) of -1.80. Similarly, Log P was identified as the primary factor influencing C_max_ for ROF N-oxide, with a SC of -0.71. Upon examining Table S2, it is observed that among all the optimized parameters, the SC value for Log P of ROF is greater than 1.0, indicating its significant impact on the C_max_ for ROF. Subsequently, the SC value was further assessed when the Log P of ROF was varied from 3.5 (as used in this study) to 3.99 (the literature value) [9]. The assessed SC value was found to be 1.11, slightly higher than 1.0.

### Validation of PBPK models for CYP3A4/1A2 inhibitors and reducers

**Fig. 3:**
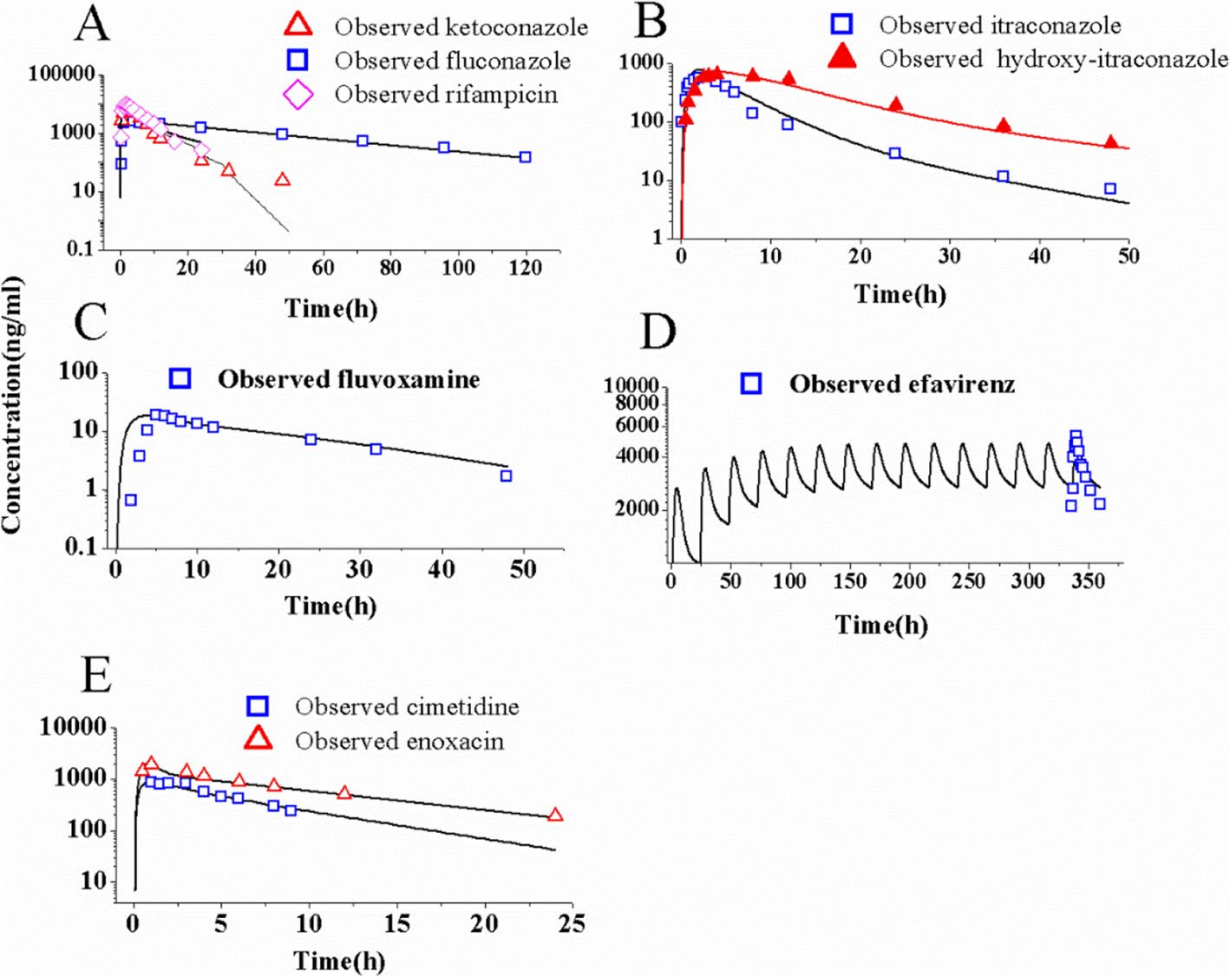
The mean predicted and observed plasma concentration-time profiles of eight CYP3A4 and CYP1A2 perpetrators. Ketoconazole, fluconazole, and rifampicin (**A**); itraconazole (**B**); fluvoxamine (**C**); efavirenz (**D**); and cimetidine and enoxacin in healthy humans (**E**)

Figure 3 presents the mean predicted PK profiles and clinically observed data for eight CYP3A4/1A2 inhibitors and reducers. The predicted and observed PK data are given in Supplementary Table S3. By comparing predictions with observations of eight perpetrators, the accuracies of PK prediction by the PBPK models of perpetrators have been verified. Table S3 shows that each ratio falls between 0.5 and 2.0. The simulations demonstrate that the PBPK models for the eight perpetrators have been successfully developed and match well with their clinically observed PK values.

### DDI simulations

**Fig. 4:**
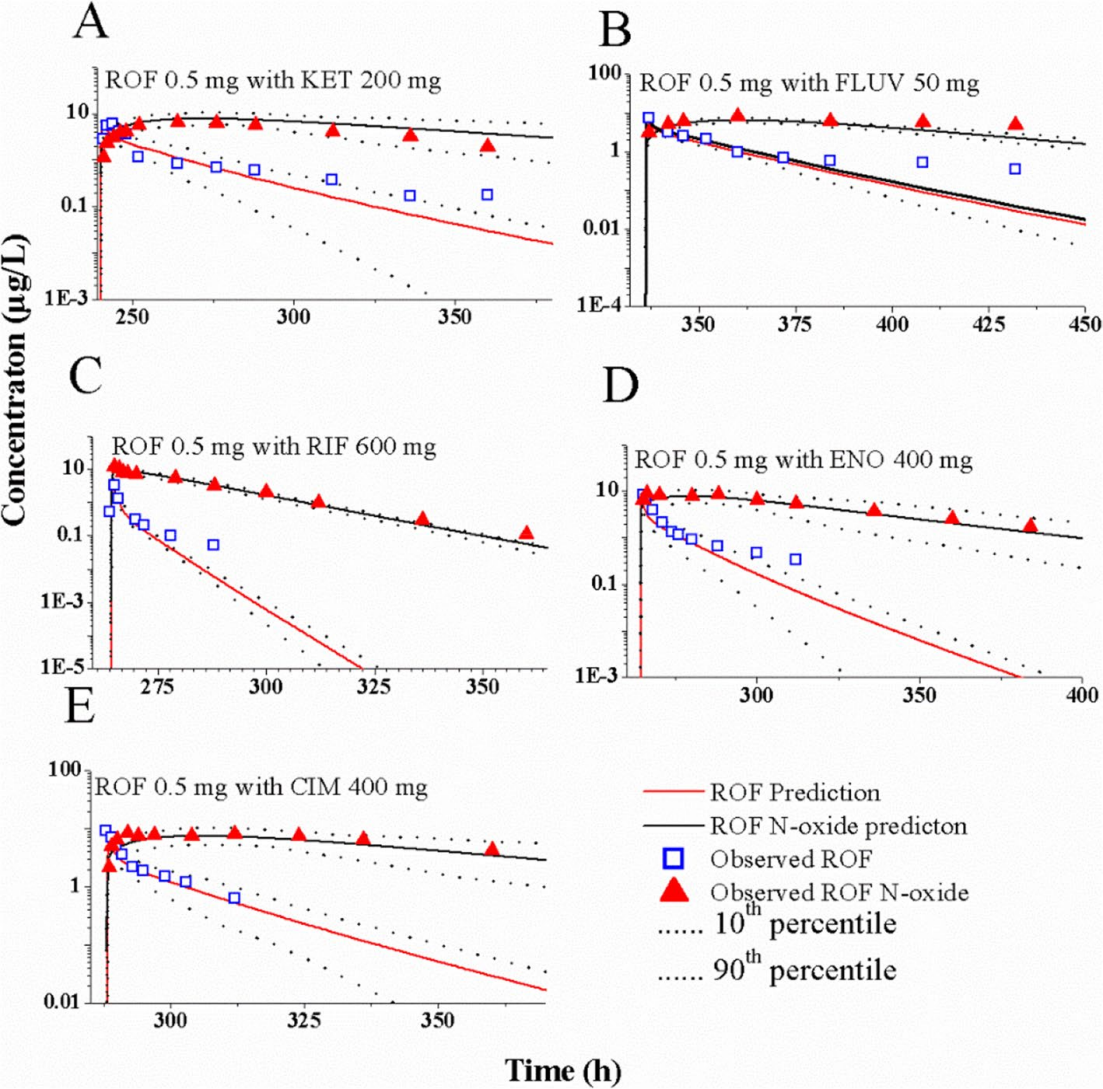
Simulations of pharmacokinetics of ROF and ROF N-oxide with ketoconazole (**A**), fluvoxamine (**B**), rifampicin (**C**), enoxacin (**D**), and cimetidine (**E**)

The DDI simulations were conducted using the PBPK model of ROF and ROF N-oxide in combination with the PBPK model of eight CYP3A4/1A2 perpetrators, respectively. Figure 4 illustrates the predicted and observed plasma concentration-time profiles in healthy individuals after the simultaneous administration of ROF with five CYP3A4 and CYP1A2 perpetrators for which DDI studies could be obtained from the papers [27, 33–36]. The DDI simulations indicate that the PBPK model aligns closely with the observed PK profiles of ROF and its metabolite, ROF N-oxide, when DDIs are present. Table 4 presents the predicted AUC, C_max_, and tPDE4i ratios of ROF and ROF N-oxide when concurrently administered with the eight perpetrators, as determined by the PBPK model. With the exception of tow C_max_ ratios of ROF with RIF (prediction: 0.63 vs. observation: 0.19) and with CIM (prediction: 1.07 vs. observation: 1.85) (Table 4), the remaining predicted ratios were very close to the observed data for five perpetrators available in the papers [27, 33–36]. To ensure the accuracy of DDI predictions, it is vital to validate the interaction parameters (K_i_, EC_max_, EC_50_) utilized in this study. This validation is necessary because variations in these parameters can be observed across different research papers. In order to validate the interaction parameters, we employed the PK variables of oral midazolam (a CYP3A4 substrate) and tizanidine (a CYP1A2 substrate) in the presence of perpetrations. Supplementary Table S4 presents the DDI predictions, indicating the reliability of the interaction parameters for the perpetrating drugs, except for the C_max_ ratios of midazolam with RIF, which exceeded 2.0. However, due to the unavailability of clinical PK data for the co-administration of ENO and CIM with tizanidine, we did not provide AUC and C_max_ ratios for tizanidine with ENO and CIM in this work.

**Table 4:** PK and PD changes of ROF and ROF N-oxide in DDIs

Perpetrators	Dosing regimens	Victims	Predicted ratios^a^			Observed ratios^a^		
			AUC	C_max_	tPDE4i^b^	AUC	C_max_	tPDE4i^b^
KET	ROF: Repeated-doses of 0.5 mg OD from days 1 to 11; KET: A single dose of 200 mg on day 11	ROF	1.43	1.07	-	1.34	1.06	NR
		ROF N-oxide	1.06	0.95		0.88	0.80	
	ROF: Single-dose of 0.5 mg OD on days 1 and 11, respectively; KET: Repeated-doses of 200 mg BID from days 8 to 20	ROF	2.07	1.18	1.34	2.01	1.23	1.10
		ROF N-oxide	1.33	0.96		1.09	0.62	
FLUV	ROF: Single-dose of 0.5 mg OD on day 15; FLU: Repeated-doses of 50 mg OD from days 8 to 21	ROF	2.29	1.16	1.62	2.56	1.12	1.59
		ROF N-oxide	1.66	0.84		1.52	0.80	
RIF	ROF: Single-dose of 0.5 mg OD on day 12; RIF: Repeated-doses of 600 mg OD from days 5 to 15	ROF	0.20	0.63	0.44	0.32	0.19	0.43
		ROF N-oxide	0.44	1.28		0.49	1.30	
ENO	ROF: Single-dose of 0.5 mg OD on days 1 and 12, respectively; ENO: Repeated-doses of 400 mg BID from days 2 to 19	ROF	1.22	1.19	1.21	1.58	1.20	1.25
		ROF N-oxide	1.02	1.12		1.20	0.86	
CIM	ROF: Single-dose of 0.5 mg OD on days 1 and 13, respectively; CIM: Repeated-doses of 400 mg BID from days 6 to 16	ROF	1.37	1.07	1.06	1.46	1.85	1.48
		ROF N-oxide	1.04	0.94		0.96	1.27	
ITR	Concomitantly used at repeated-doses of ITR 200 mg OD, FLU 150 mg OD, EFA 600 mg OD, respectively, and ROF 0.5 mg OD on day14	ROF	2.14	1.16	1.40	NR		NR
		ROF N-oxide	1.22	0.70				
FLU		ROF	1.89	1.18	1.16			
		ROF N-oxide	1.12	0.92				
EFA		ROF	0.28	0.64	0.58			
		ROF N-oxide	0.60	1.16				

^a^Calculated by ratio of PK variables with and without perpetrators

^b^Calculated using Eq. (4)

Of these DDI simulations, ratios of ROF were either more than 2-fold or less than 0.5-fold occurred when co-administered with KET, FLUV, RIF, ITR, and EFA at repeated-doses. In contrast, the ratios of ROF N-oxide was less than 0.5-fold (0.44-fold) only when concurrently used with RIF. Similarly, the changes in tPDE4i ratios mirrored PK changes of ROF N-oxide, likely due to the 90% contribution of tPDE4i provided by ROF N-oxide.

**Fig. 5:**
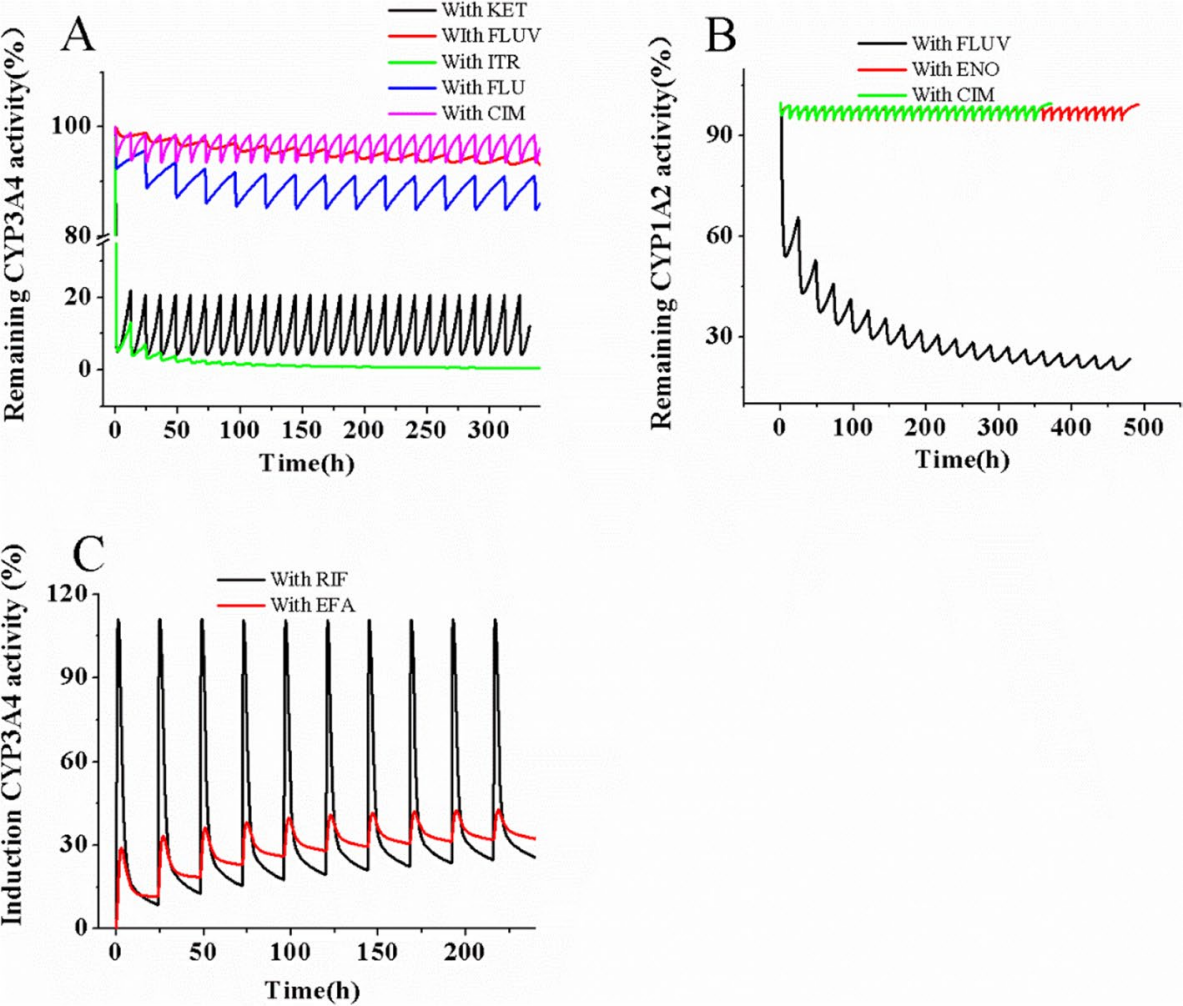
Effect of eight perpetrators on hepatic CYP3A4 and CYP1A2 activity Remaining CYP3A4 activity after inhibition by KET, FLUV, ITR, FLU, and CIM, respectively (**A**). Remaining CYP1A2 activity after inhibition by FLUV, ENO, and CIM, respectively (**B**). Increased CYP3A4 activity after induction by RIF and EFA, respectively (**C**)

 The liver’s CYP3A4 and CYP1A2 activities were evaluated over time during DDIs, and the simulations are depicted in Fig. 5. The maximum inhibition of CYP3A4 occurred with ITR, while FLUV caused the highest inhibition of CYP1A2. Despite RIF exhibiting a stronger maximum induction effect on CYP3A4 compared to EFA, the trough induction effect of RIF was lower than that of EFA. This may explain the minor difference in AUC ratios of ROF and ROF N-oxide with two inducers.

### Dosage adjustment recommendations based on the DDI simulations

In general, clinical dosing regimens should be modified when the plasma AUC ratio increases or decreases by more than 2-fold in the presence of DDIs [37]. However, the clinical exposure-response for efficacy and safety suggested that the maximum tolerated dose of ROF is identified as 0.5 mg OD [9]. Moreover, since ROF N-oxide contributes 90% inhibition of iPDE4i, dosage regimen for ROF cannot solely rely on the AUC changes of ROF itself in DDIs. Therefore, the following criteria are defined for dosing regimens of ROF: ➀ when both AUC ratios of ROF and ROF N-oxide are within the range of 0.8–1.25 in DDIs, ROF does not require dose adjustment; ➁ when changes in AUC ratios of ROF and ROF N-oxide are between 0.5 and 2.0, but outside the range of 0.8–1.25, in DDIs, ROF may be used with caution; ➂ when changes in AUC ratios of ROF or ROF N-oxide exceed 2-fold or decrease to less than 0.5-fold in DDIs, co-administration of ROF should be prohibited, rather than reducing the dose.

**Fig. 6:**
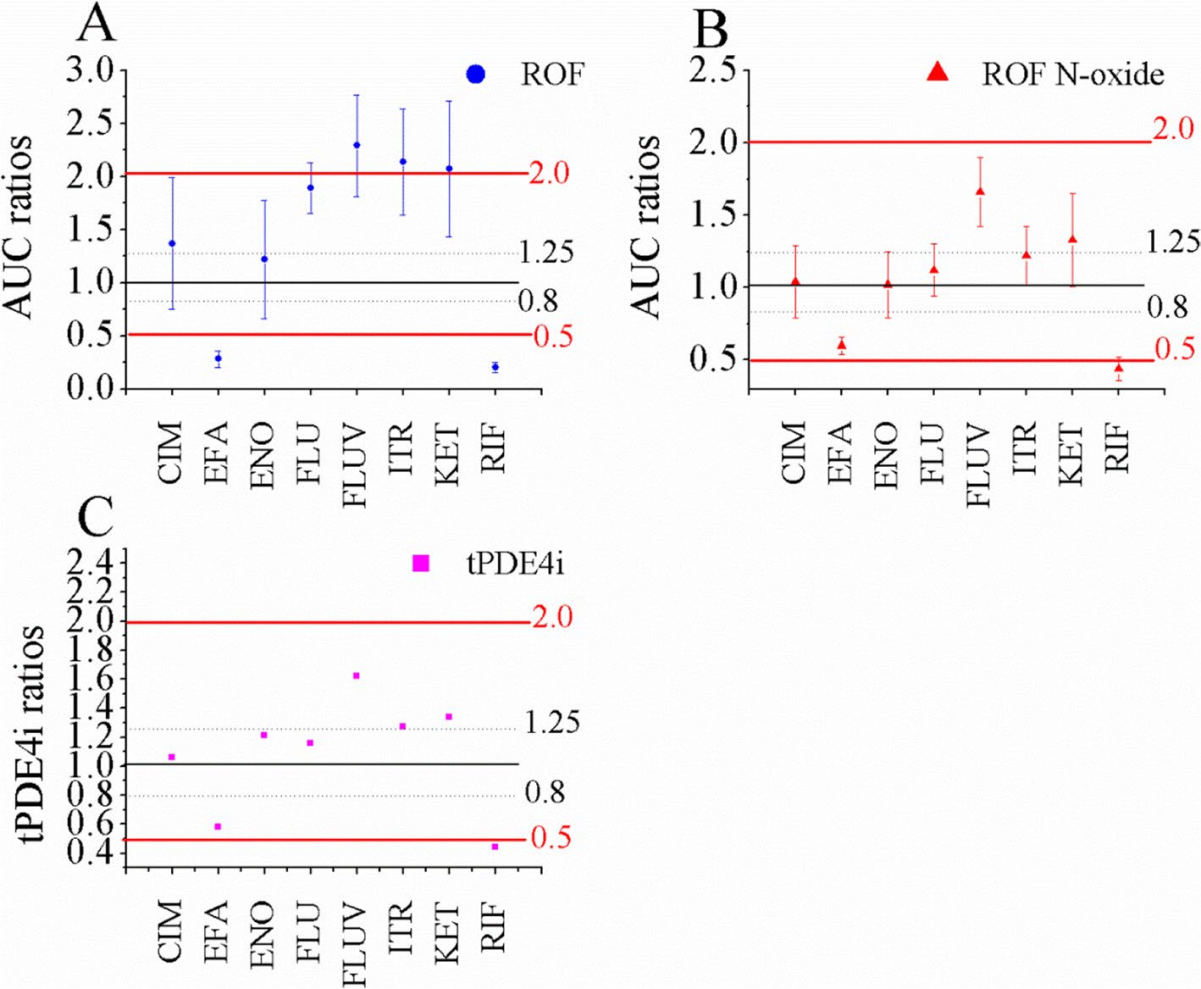
Simulations of AUC ratios and tPDE4i of ROF and ROF N-oxide in the DDIs. AUC ratios change of ROF (**A**) and ROF N-oxide (**B**), and tPDE4i change (**C**) with eight CYP3A3/1A2 perpetrators. Data were shown as geometric mean values and 90% CI

 As shown in Fig. 6, only when co-administered with ENO, the average AUC ratios of ROF fell between 0.8 and 1.25, indicating no significant differences in DDIs. However, its 90% confidence interval (CI) was still outside the range of 0.8–125. Additionally, AUC ratios of ROF increased or decreased by more than 2-fold in five DDI cases (with KET, ITR, FLUV, RIF, and EFA).In contrast, average AUC ratios of ROF N-oxide were all between 0.8–1.25 after co-administration of ROF with four perpetrators. Furthermore, AUC ratios of ROF N-oxide reduced by more than 2-fold only when co-administered with RIF. This suggests ROF N-oxide is less influenced by DDIs compared to ROF.

According to the changes in AUC ratio of ROF, the dosage of ROF should be reduced to 0.25 mg when concurrently used with KET, ITR and FLUV. However, considering clinical efficacy (the AUC ratios of ROF N-oxide and tPDE4i ratios are less than 2.0-fold change), it may be a suitable option to cautiously co-administer ROF with the three perpetrators with caution .Although the average AUC ratios of ROF and ROF N-oxide were all between 0.5- and 2-fold, it was still suggested that co-administration with the three perpetrators (CIM, ENO, and FLU) should use with caution based on clinical maximum toleration. Additionally, the DDI simulations suggest that ROF should be recommended to avoid continuous co-administration of ROF with RIF and EFA. The dosage recommendations in DDIs by the PBPK model are in good agreement with the clinical dosing proposals [8].

## Discussion

In this work, the PBPK model of ROF and ROF N-oxide was successfully developed and was able to accurately predict the plasma AUC and C_max_ for both healthy subjects and COPD patients. Moreover, the PBPK model accurately predicted the ratio change in AUC, C_max_, and tPDE4i of ROF and ROF N-oxide when concurrently administered with eight CYP3A4/1A2 perpetrators. The prediction accuracy of the PBPK model was supported by the multiple clinical PK studies [23–27, 33–36] (Figs. 2 and 4, Tables 3 and 4). Furthermore, dosage recommendations of ROF were proposed when co-administered with the eight CYP3A4/1A2 perpetrators based on AUC ratios. Notably, this is the first study to develop the PBPK model for ROF and simulate the PK and PD (i.e., tPDE4i) changes in the presence DDIs.

The two parameters, distribution method and K_p_ caling (K_Ins,p_ and K_Inc,p_), are associated with drug tissue distribution in the PBPK model. In PK-Sim, tissue distribution is determined using five methods: Rodgers and Rowland, PK-Sim standard, Schmitt, Poulin and Theil, and Berezhkovskiy. On the other hand, cellular permeability is calculated using two methods: PK-Sim standard and Charge dependent Schmitt. To better agree with the observed concentration-time profiles of ROF and ROF N-oxide, the distribution calculation in the PBPK model was optimized using the parameter identification module in PK-Sim. The identified method for tissue distribution calculation was Rodgers and Rowland, while the PK-Sim standard method was selected for cellular permeability calculation. Additionally, to improve agreement with the time-concentration profiles, the K_Ins,p_ scale was optimized to 5.0 specifically for ROF. Furthermore, the sensitivity analysis presented in Table S2 demonstrated that among the optimized parameters, Log P of ROF was identified as the most sensitive parameter for ROF C_max_. However, it was found that within the range of Log P values from 3.99 to 3.5, it only had a slight influence on the ROF C_max_. Based on this observation, it can be concluded that these optimized parameters fall within an acceptable range.

KET, ITR, and FLU are competitive strong and moderate inhibitors of CYP3A4. RIF and EFA are strong and moderate inducers of CYP3A4. ENO is competitive inhibitors of CYP1A2; FLUV and CIM are dual inhibitors of CYP3A4 and CYP1A2. Although *in vitro* K_i_ values of ENO and CIM against CYP3A4 and CYP1A2 are relatively higher compared to other inhibitors, they both have higher free plasma concertation (0.72 and 0.84, Supplementary Table S1). Therefore, *in vivo*, they could result in a moderate DDI. These eight perpetrators were selected to simulate the PK and PD effects on ROF and ROF N-oxide. Previous studies have demonstrated that a reasonable value for k_deg_ of CYP enzymes for accurate *in vivo* prediction of multiple drugs is 0.03 h [22]. Hence, a k_deg_ value of 0.03 h^−1^ was used in present PBPK model. While some papers have shown that the activity of CYP enzymes differs between the gut and liver [38], the current PBPK model did not incorporate different CYP1A2/3A4 activity. However, different k_deg_ values were set for gut and liver in the present PBPK model (Table 1). Moreover, the induction parameters (E_max_ and EC_50_) of RIF showed wide variability among different experimental papers [39, 40]. To minimize this variation, the values from the latest PBPK model paper were used [28].

There are still limitations to the present PBPK model. Firstly, in the simulation, both CYP3A4 CL_int,u_ and CYP1A2 CL_int,u_ were reduced by the same ratio in patients with COPD compared to healthy subjects. However, it is important to note that this assumption was made without specific empirical data supporting the exact magnitude of the reduction at present. Moreover, this PBPK approach does not take into consideration other physiological differences in COPD patients as well. A second challenge is the lack of clinical validation for DDI simulations with ITR, FLU, and EFA.

## Conclusions

In summary, the PBPK model successfully predicted the clinical PK and PD of ROF and ROF N-oxide in both healthy subjects and COPD patients. Additionally, the model accurately predicted DDI outcomes in combination with CYP3A4 and CYP1A2 perpetrators. Furthermore, based on the PBPK model, a dosage adjustment strategy for ROF was proposed when co-administered in DDIs.

### Supplementary Information

**Additional file 1: Supplementary Table S1.** Inputting parameters used for the PBPK models of CYP3A4 and CYP1A2 perpetrators in DDI simulations. **Supplementary Table S2.** Modelling parameters sensitivity analysis. **Supplementary Table S3.** The mean observed and predicted PK parameters for the eight perpetrators based on their respective PBPK model. **Supplementary Table S4.** C_max_ and AUC ratios between prediction and observation for midazolam and tizanidine with CYP3A4 andCYP1A2 perpetrators.

## Data Availability

All data analyzed or generated in this study can be obtained from this manuscript and supplementary tables.
