# Supplementary material for: Prediction of drug–drug interactions between roflumilast and CYP3A4/1A2 perpetrators using a physiologically-based pharmacokinetic (PBPK) approach
Source: BMC Pharmacol Toxicol. 2024 Jan 2;25:4. doi: 10.1186/s40360-023-00726-2 (PMC10762902; doi:10.1186/s40360-023-00726-2)
Supplement: Supplementary file 1 — Additional file 1: Supplementary Table S1. Inputting parameters used for the PBPK models of CYP3A4 and CYP1A2 perpetrators in DDI simulations. Supplementary Table S2. Modelling parameters sensitivity analysis. Supplementary Table S3. The mean observed and predicted PK parameters for the eight perpetrators based on their respective PBPK model. Supplementary Table S4. Cmax and AUC ratios between prediction and observation for midazolam and tizanidine with CYP3A4 andCYP1A2 perpetrators. [file 40360_2023_726_MOESM1_ESM.docx]

**Supplementary Files**

**Supplementary Table S1** Inputting parameters used for the PBPK models of CYP3A4 and CYP1A2 perpetrators in DDI simulations

**Supplementary Table S2** Modelling parameters sensitivity analysis

**Supplementary Table S3** The mean observed and predicted PK parameters for the eight perpetrators based on their respective PBPK model

**Supplementary Table S4** C_max_ and AUC ratios between prediction and observation for midazolam and tizanidine with CYP3A4 andCYP1A2 perpetrators

**Supplementary Table S1** Inputting parameters used for the PBPK models of CYP3A4 and CYP1A2 perpetrators in DDI simulations

| Property | Values | | | | | | | |
| --- | --- | --- | --- | --- | --- | --- | --- | --- |
|  | Ketoconazole^a^ | Itraconazole/ Hydroxy-itraconazole^b^ | Fluconazole^a^ | Fluvoxamine^a^ | Rifampicin^a^ | Efavirenz^a^ | Cimetidine^c^ | Enoxacin^d^ |
| MW(g·mol^-1^) | 531.4 | 705.6/721.7 | 306.3 | 318.34 | 822.94 | 315.68 | 252.34 | 320.32 |
| pKa | 6.51(base) | 3.7/2.53,4.91(base) | 2.03(neutral) | - | 1.7(acid), 7.9(base) | 10.1(acid) | 6.9(base) | 6.3（acid），8.7（base） |
| Log P | 2.67(@pH7.4) | 4.2/3.5 | 0.83 | 3.57 | 2.3 | 3.44 | 2.0 | 1.9 |
| Solubility(μg/mL) | 6.93(@pH6.5) | 5.4(@pH1.2)/- | 6900 | 14660 | 2.8(@pH7.5) | 39.9 | - | - |
| P_eff_ (🞨10^-5^ cm⋅s^-1^) | 1.24 | - | 21.3 | 0.046 | 1.24 | 2.97 | - | 1.5 |
| P_app_ (🞨10^-6^ cm⋅s^-1^) | - | 57.1/- | - | - | - | - | 22.1 | - |
| f_up_ | 0.015 | 0.016/0.021 | 0.11 | 0.23 | 0.17 | 0.0059 | 0.836 | 0.72 |
| Rbp | 0.59 | 0.58/0.58 | 0.85 | 5.5 | 0.89 | 0.63 | 1.0 | 0.77 |
| CL_H_（mL/min/kg） | - | - | - | - | - | - | 3.0 | 226.8 |
| UGT2B7 CL_int_（L/min） | - | - | 0.008 | - | - | - | - | - |
| CYP3A4 V_max_ (pmol/min/pmol) | 8.0 | 0.65/0.05 | - | - | - | 0.16 | - | - |
| CYP3A4 K_m_ (μM) | 15.0 | 0.039/0.027 | - | - | - | 23.5 | - | - |
| CYP3A5 V_max_ (pmol/min/pmol) | - | - | - | - | - | 0.6 | - | - |
| CYP3A5 K_m_ (μM) | - | - | - | - | - | 19.1 | - | - |
| UGT1A1 V_max_ (pmol/min/pmol) | 9.37 | - | - | - | - | - | - | - |
| UGT1A1 K_m_ (μM) | 22.3 | - | - | - | - | - | - | - |
| CYP1A2 CL_int_ (pmol/min/mg) | - | - | - | 2.0 | - | - | - | - |
| CYP1A2 V_max_ (pmol/min/pmol) | - | - | - | - | - | 0.60 | - | - |
| CYP1A2 K_m_ (μM) | - | - | - | - | - | 8.3 | - | - |
| CYP2A6 V_max_ (pmol/min/pmol) | - | - | - | - | - | 1.0 | - | - |
| CYP2A6K_m_ (μM) | - | - | - | - | - | 7.7 | - | - |
| CYP2B6 V_max_ (pmol/min/pmol) | - | - | - | - | - | 3.5 | - | - |
| CYP2B6K_m_ (μM) | - | - | - | - | - | 6.4 | - | - |
| CYP2D6 V_max_ (pmol/min/pmol) | - | - | - | 0.10 | - | - | - | - |
| CYP2D6K_m_ (μM) | - | - | - | 76.3 | - | - | - | - |
| AADAC V_max_ (μM/min) | - | - | - | - | 9.87 | - | - | - |
| AADAC K_m_ (μM) | - | - | - | - | 195.10 | - | - | - |
| P-gp V_max_ (μM/min) | - | - | - | - | 0.036 | - | - | - |
| P-gp K_m_ (μM) | - | - | - | - | 55.0 | - | - | - |
| OATP1B1 V_max_ (μM/min) | - | - | - | - | 0.086 | - | - | - |
| OATP1B1K_m_ (μM) | - | - | - | - | 1.5 | - | - | - |
| CL_R_(L/h) | - | f_up_🞨GFR | | | | | |  |
| GFR fraction | - | 1.0 | 0.2 |  | 1.0 | 1.0 | 1.0 | 1.0 |
| K_p_ scale | - | 0.6/2.0 | - | - | 3.0 | - | - | - |
| Partition coefficients | Rodgers and Rowland | | | | Schmitt | | Rodgers and Rowland | |
| Cellular permeabilities | PK-Sim Standard | | | | PK-Sim Standard | | PK-Sim Standard | |
| Weibull time (min) | 120 | 110 | 120 | 120 | 10 | 240 | 120 | 15 |
| Weibull shape | 0.92 | 0.92 | 0.92 | 0.92 | 0.92 | 0.92 | 0.92 | 0.92 |

^a^: The modeling parameters were built in the OSP library of PK-Sim.

^b^: The modeling parameters were taken from the reference S1.

^c^: The modeling parameters were taken from the reference S2-S3.

^d^: The modeling parameters were taken from the reference S4-S7.

The clinical observed PK data were taken from the references S8(ketoconazole),S1 (itraconazole), S9(fluconazole), S10(Fluvoxamine),S11(rifampicin), S12 (efavirenz), S13(cimetidine), , S6(enoxacin).

[S1] Chen Y, Ma F, Lu T, et al. Development of a physiologically based pharmacokinetic model for itraconazole pharmacokinetics and drug–drug interaction prediction[J]. Clinical pharmacokinetics, 2016, 55(6): 735-749.

[S2] Jeong Y S, Balla A, Chun K H, et al. Physiologically-based pharmacokinetic modeling for drug-drug interactions of procainamide and N-acetylprocainamide with cimetidine, an inhibitor of rOCT2 and rMATE1, in rats[J]. Pharmaceutics, 2019, 11(3): 108.

[S3] Ikeda K, Ueda C, Yamada K, et al. Carrier-mediated placental transport of cimetidine and valproic acid across differentiating JEG-3 cell layers[J]. Die Pharmazie-An International Journal of Pharmaceutical Sciences, 2015, 70(7): 471-476.

[S4] Yang L P, Zhou Z W, Chen X W, et al. Computational and in vitro studies on the inhibitory effects of herbal compounds on human cytochrome P450 1A2[J]. Xenobiotica, 2012, 42(3): 238-255.

[S5] Fang J Y, Lin H H, Chen H I, et al. Development and evaluation on transdermal delivery of enoxacin via chemical enhancers and physical iontophoresis[J]. Journal of controlled release, 1998, 54(3): 293-304.

[S6] Nix D E, Schultz R W, Frost R W, et al. The effect of renal impairment and haemodialysis on single dose pharmacokinetics of oral enoxacin[J]. Journal of Antimicrobial Chemotherapy, 1988, 21(suppl_B): 87-95.

[S7] Chang T, Black A, Dunky A, et al. Pharmacokinetics of intravenous and oral enoxacin in healthy volunteers[J]. Journal of Antimicrobial Chemotherapy, 1988, 21(suppl_B): 49-56.

[S8] Daneshmend T K, Warnock D W. Clinical pharmacokinetics of ketoconazole[J]. Clinical pharmacokinetics, 1988, 14: 13-34.

[S9] Al-Mahroos M I A, Al-Tamimi D J J, Al-Tamimi Z J J, et al. Clinical pharmacokinetics and bioavailability study between generic and branded fluconazole capsules[J]. Journal of Advanced Pharmacy Education & Research| Jan-Mar, 2021, 11(1).

[S10] Spigset O, Carleborg L, Hedenmalm K, et al. Effect of cigarette smoking on fluvoxamine pharmacokinetics in humans[J]. Clinical Pharmacology & Therapeutics, 1995, 58(4): 399-403.

[S11] Hanke N, Frechen S, Moj D, et al. PBPK models for CYP3A4 and P‐gp DDI prediction: a modeling network of rifampicin, itraconazole, clarithromycin, midazolam, alfentanil, and digoxin[J]. CPT: pharmacometrics & systems pharmacology, 2018, 7(10): 647-659.

[S12] Ji P, Damle B, Xie J, et al. Pharmacokinetic interaction between efavirenz and carbamazepine after multiple‐dose administration in healthy subjects[J]. The Journal of Clinical Pharmacology, 2008, 48(8): 948-956.

[S13] Somogyi A, Gugler R. Clinical pharmacokinetics of cimetidine[J]. Clinical pharmacokinetics, 1983, 8: 463-495.

**Supplementary Table S2**  Modelling parameters sensitivity analysis

| Modelling parameters | SC values | | | |
| --- | --- | --- | --- | --- |
|  | AUC for ROF | C_max_ for ROF | AUC for ROF N-oxide | C_max_ for ROF N-oxide |
| LogP | -0.85 | -1.80 | -0.54 | -0.71 |
| CYP3A4 CL_int,u_ | -0.82 | -0.28 | -0.30 | -0.28 |
| CYP1A2 CL_int,u_ | -0.30 | -0.07 | **-** | **-** |
| K_Ins,p scale_ | - | - | 0.18 | **-** |

-: SC value <0.1.

**Supplementary Table S3** The mean observed and predicted PK parameters for the eight perpetrators based on their respective PBPK model

| Perpetrators | Parameters | Predicted | Observed | Predicted/Observed |
| --- | --- | --- | --- | --- |
| Ketoconazole | Cmax (μg·mL^-1^) | 7289.1 | 6240.0 | 1.17 |
|  | AUC0-48 (μg·h·mL^-1^) | 5235.9 | 4687.2 | 1.12 |
|  | Tmax (h) | 1.7 | 1.5 | 1.13 |
| Itraconazole | C_max_ (ng·mL^-1^) | 765.6 | 556.0 | 1.38 |
|  | AUC_0-48_ (ng·h·mL^-1^) | 5511.3 | 4500.5 | 1.22 |
|  | T_max_ (h) | 1.9 | 2.0 | 0.95 |
| Hydroxy- Itraconazole | C_max_ (ng·mL^-1^) | 723.4 | 639.0 | 1.13 |
|  | AUC_0-48_ (ng·h·mL^-1^) | 11676.6 | 13128.9 | 0.89 |
|  | T_max_ (h) | 3.5 | 4.0 | 0.88 |
| Fluconazole | C_max_ (ng·mL^-1^) | 2820 | 2649 | 1.06 |
|  | AUC_0-48_ (μg·h·mL^-1^) | 109.0 | 106.5 | 1.02 |
|  | T_max_ (h) | 1.5 | 2.5 | 0.6 |
| Fluvoxamine | C_max_ (ng·mL^-1^) | 18.0 | 18.5 | 0.97 |
|  | AUC_0-48_ (ng·h·mL^-1^) | 389.4 | 331.9 | 1.17 |
|  | T_max_ (h) | 3.75 | 5.0 | 0.75 |
| Rifampicin | C_max_ (ng·mL^-1^) | 10977.2 | 9540.0 | 1.15 |
|  | AUC_0-24_ (ng·h·mL^-1^) | 63191.4 | 62861.3 | 1.01 |
|  | T_max_ (h) | 1.2 | 1.5 | 0.80 |
| Efavirenz | C_max_ (ng·mL^-1^) | 4791.4 | 5240.0 | 0.91 |
|  | AUC_336-360_ (ng·h·mL^-1^) | 11284.6 | 11644.5 | 0.97 |
|  | T_max_ (h) | 340 | 341 | 1.00 |
| Cimetidine | C_max_ (ng·mL^-1^) | 884.8 | 846.0 | 1.05 |
|  | AUC_0-24_ (ng·h·mL^-1^) | 6488.2 | 4676.2 | 1.39 |
|  | T_max_ (h) | 0.90 | 1.0 | 0.90 |
| Enoxacin | C_max_ (ng·mL^-1^) | 6.1 | 5.9 | 1.03 |
|  | AUC_0-24_ (μg·h·mL^-1^) | 14.5 | 15.8 | 0.92 |
|  | T_max_ (h) | 0.75 | 1.0 | 0.75 |

**Supplementary Table S4** C_max_ and AUC ratios between prediction and observation for midazolam and tizanidine with CYP3A4 andCYP1A2 perpetrators

| Perpetrators | Victims | Dosing regimens | Observation | | Prediction | | Prediction/Observation | |
| --- | --- | --- | --- | --- | --- | --- | --- | --- |
|  |  |  | C_max_ | AUC | C_max_ | AUC | C_max_ ratios | AUC ratios |
| KET | Midazolam | 6 mg midazolam with 200 mg KET were administered. The first dose of KET was administered 12 h before midazolam dose and subsequent doses were administered every 12 h for three doses. ^[S14]^ | 4.21 | 15.1 | 3.21 | 12.51 | 0.76 | 0.83 |
| ITR |  | A single dose of 7.5 mg midazolam were administered, respectively, on days 1and 6. 200 mg OD of ITR was administered for consecutive 6days and fist dose was administered 12 h after the first midazolam ^[S15]^. | 1.80 | 6.64 | 2.03 | 5.73 | 1.13 | 0.86 |
| FLU |  | 7.5 mg midazolam on days 1and 6 were administered, respectively. 400 mg OD of FLU was administered on the first day and then 200 mg OD for consecutive 5 days. The first dose of FLU was administered 12 h after the first midazolam ^[S15]^. | 1.74 | 3.60 | 1.55 | 3.89 | 0.89 | 1.08 |
| FLUV | Midazolam | 50 mg BID of FLUV were dosed for consecutive 5 days, and then increased to100 mg BID for consecutive 6 days. 10 mg midazolam was dosed 1 h after the last dose of FLUV ^[S16]^. | 1.40 | 1.38 | 1.26 | 1.59 | 0.90 | 1.15 |
|  | Tizanidine | Repeated-doses of100 mg FLU was administered for 4 days. On day 4, a single oral dose of 4 mg tizanidine was administered ^[S17]^. | 12.1 | 32.7 | 17.7 | 29.5 | 1.46 | 0.90 |
| ENO | Tizanidine | 4 mg OD of tizanidine and 400 mg BID of ENO were co-administered for 7days | - | - | 1.22 | 1.19 | - | - |
| CIM | Midazolam | 15 mg midazolam was administered for 1 week, and with the addition of a single dose of 400 mg of CIM 2 h before the last dose ^[S18]^. | 1.37 | 1.36 | 1.34 | 1.16 | 0.98 | 0.85 |
|  | Tizanidine | 4 mg OD of tizanidine and 400 mg BID of CIM were co-administered for 7days | - | - | 1.36 | 1.28 | - | - |
| RIF | Midazolam | 600 mg OD of RIF was dosed for consecutive 5 days, and then increased to100 mg BID for consecutive 6 days. A single dose of 15 mg midazolam was dosed 17 h after the last dose of RIF ^[S19]^. | 0.06 | 0.04 | 0.19 | 0.08 | 3.17 | 2.00 |
| EFA | Midazolam | 4 mg midazolam was adminisered 12 h after the last dose of EFA. 400 mg OD of EFA was administered ^[S20]^. | 0.73 | 0.59 | 0.84 | 0.65 | 1.15 | 1.10 |

-:Not reported.

[S14] Tsunoda S M, Velez R L, von Moltke L L, et al. Differentiation of intestinal and hepatic cytochrome P450 3A activity with use of midazolam as an in vivo probe: effect of ketoconazole[J]. Clinical pharmacology & therapeutics, 1999, 66(5): 461-471.

[S15] Olkkola K T, Ahonen J, Neuvonen P J. The effect of the systemic antimycotics, itraconazole and fluconazole, on the pharmacokinetics and pharmacodynamics of intravenous and oral midazolam[J]. Anesthesia & Analgesia, 1996, 82(3): 511-516.

[S16] Lam Y W F, Alfaro C L, Ereshefsky L, et al. Pharmacokinetic and pharmacodynamic interactions of oral midazolam with ketoconazole, fluoxetine, fluvoxamine, and nefazodone[J]. The Journal of Clinical Pharmacology, 2003, 43(11): 1274-1282.

[S17]Granfors M T, Backman J T, Neuvonen M, et al. Fluvoxamine drastically increases concentrations and effects of tizanidine: a potentially hazardous interaction[J]. Clinical Pharmacology & Therapeutics, 2004, 75(4): 331-341.

[S18] Salonen M, Aantaa E, Aaltonen L, et al. Importance of the interaction of midazolam and cimetidine[J]. Acta pharmacologica et toxicologica, 1986, 58(2): 91-95.

[S19] Backman J T, Olkkola K T, Neuvonen P J. Rifampin drastically reduces plasma concentrations and effects of oral midazolam[J]. Clinical pharmacology & therapeutics, 1996, 59(1): 7-13.

[S20] Mikus G, Heinrich T, Bödigheimer J, et al. Semisimultaneous midazolam administration to evaluate the time course of CYP3A activation by a single oral dose of efavirenz[J]. The Journal of Clinical Pharmacology, 2017, 57(7): 899-905.
